# Construction and application of a high precision 3D simulation model for geomechanics of the complex coal seam

**DOI:** 10.1038/s41598-021-00709-5

**Published:** 2021-11-01

**Authors:** Lijuan Zhao, Meichen Zhang, Xin Jin

**Affiliations:** 1 School of Mechanical Engineering, Liaoning Technical University, Fuxin, China; 2 The State Key Lab of Mining Machinery Engineering of Coal Industry, Liaoning Technical University, Fuxin, China; 3 Liaoning Province Large Scale Industrial and Mining Equipment Key Laboratory, Fuxin City, 123000 Liaoning Province China

**Keywords:** Energy science and technology, Engineering

## Abstract

The high-precision 3D simulation model for geomechanics of a complex coal seam is the necessary premise for the research on intelligent shearer and unmanned mining. However, at present, a simulation model for geomechanics of a complex coal seam generally has the problems of simplifying complex geological structures and low accuracy for structures. In order to meet the needs of a coal seam simulation model in the mining process of an intelligent shearer, it is necessary to optimize the simplified model of a coal seam. Therefore, based on a 3D simplified simulation model constructed with discrete element technology, the complex coal seam application plug-in was compiled with the help of an Application Program Interface. Moreover, according to the geological characteristics, new attributes were added to the structures to complete the construction of the model of a complex coal seam. Finally, the model was verified with laboratory experiments. The results showed that the high-precision 3D simulation model for geomechanics of a complex coal seam effectively improved the accuracy of the modeling. The real-time transmission and the real-time sharing of multi-source data were realized by considering the 3D simulation model for geomechanics of a complex coal seam as the core. Additionally, the purpose of the real-time sensing of the coal cutting state was achieved in order to lay the foundation for the realization of unmanned mining.

## Introduction

The achievement of unmanned mining is the frontier technology in the field of coal mining around the world, and the intelligent and efficient cutting of a shearer is the premise of unmanned mining^1,2^. A spiral drum is the working mechanism of a shearer, that undertakes the two functions of falling coal and loading coal. For a coal seam with complex occurrence conditions, the efficient cutting of a spiral drum is a complex evolution process with the characteristics of non-equilibrium, non-linearity, time-variance and strong coupling^3,4^, which is the result of multi factor coupling and which directly affects the dynamic behavior of the shearer. With the development of computer technology and the concept of digital mines and intelligent mines, more and more attention has been paid to the modeling method of the approximate representation of the objective entity of complex coal seam based on virtual prototype technology^5,6^. Therefore, based on the actual geological conditions of a mining area and the physical and mechanical characteristics of coal and rock, the establishment of the 3D simulation model for geomechanics of a coal mining face is the basis of the accurate identification and control of the cutting state of an intelligent shearer. Many scholars have carried out research in this area. Liu et al. proposed the multi-attribute dynamic modeling method for a transparent working face, that integrated the characteristics of multi-source heterogeneous data, a multi-attribute data fusion algorithm, dynamic visual modeling, and other technologies to produce the progressive transparency of a working face^7^. In order to improve the local accuracy of a coal seam 3D model, Zhang et al. proposed dynamic fine correction technology to produce the limited transparency of a working face^8^. Mao et al. proposed solutions for the key technologies of transparent mines, such as coal face modeling specification, the storage and integration of multi-source spatio-temporal data, and the 3D visualization of massive data, which had guiding significance for intelligent coal mining^9^. Sun et al. modified a 3D geological model dynamically to improve the accuracy of the model and made it more realistic to reflect the production situation^10^. Li et al. constructed the surface model of an irregular triangulation network by using the contour line of a coal seam floor, calculated the thickness value of each point in tin with Kriging interpolation, and then mapped the coal seam roof surface and finally formed the coal seam 3D model ^11^. Zhao et al. used the discrete element method to define coal and rock properties, generated the coal and rock particle group through the particle factory in EDEM, and finally formed the 3D simulation model of a coal wall^12^. Zhu et al. proposed a web-based 3D coal seam modeling scheme, and they combined this scheme with the regular grid method and the adaptive differential evolution Kriging spatial interpolation algorithm to build a digital elevation model for a coal seam surface^13^. According to the characteristics of a coal seam, Zhou et al. used 3D modeling software to build a 3D solid model of coal and rock. Through the interface between Pro/E and ANSYS, the unit type and the material type of the coal rock model were set in the finite element software, which provided a simulation model for the study of power transmission in the process of coal breaking with complex coal seam conditions^14^.

The key to building a 3D simulation model for geomechanics of a complex coal seam is to improve the accuracy of the model. The problems existing in the previous modeling methods, such as the improper simplification of the occurrence conditions of a coal seam working face, ignoring geological structures such as faults, folds, and hard nodules, the limitations of different modeling methods, and different use standards for different modeling software programs, would lead to large errors in the actual application of coal seam state information feedback. Additionally, it is difficult to achieve the accurate identification and decision-making control of a shearer cutting state.

Based on the above analysis and combined with many years of research on the virtual prototype simulation model of our research group, the author considered the coal seam discrete element 3D simplified simulation model as the basis of this study^15,16^, and with the help of an application program interface^17–19^, according to the occurrence conditions of a coal seam working face. An application plug-in was compiled, the particle factory and the contact model in the EDEM were customized, and the secondary development of a coal seam 3D simulation model was carried out. In order to integrate more information about the coal seam geological conditions, the inclusions, fault, gangue rock formation, roof, and floor structure were added to the coal seam 3D simulation model. Taking the 3D simulation model for geomechanics of a complex coal seam as the core, a multi-domain coupling collaborative real-time simulation platform with data sharing and feedback was built. This could not only improve the accuracy of the 3D simulation model for geomechanics of a coal seam but also meet the needs of intelligent control and unmanned mining.

## Key technology for constructing the high-precision 3D simulation model for geomechanics of a complex coal seam

### Modeling technology of irregular granular inclusions

**Figure 1:**
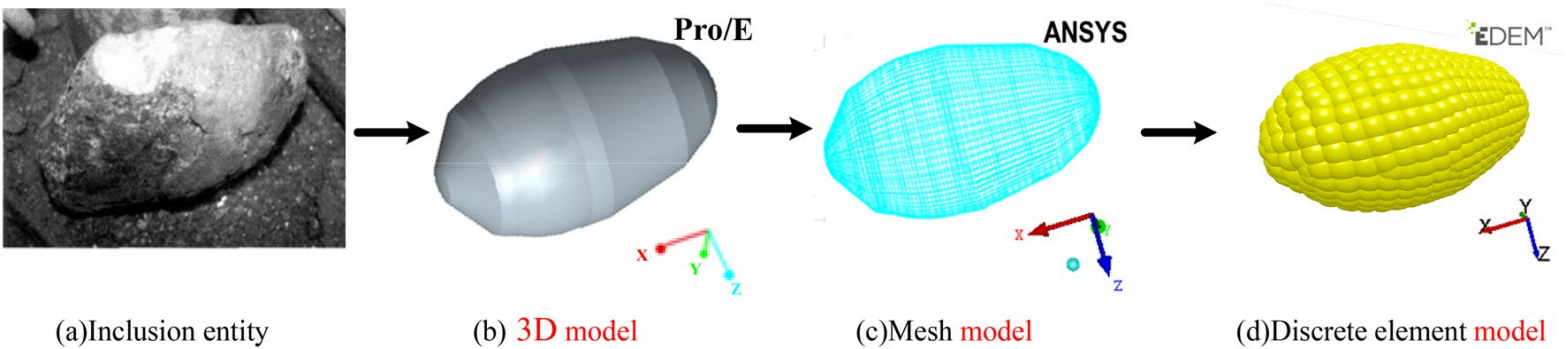
Evolution process of inclusion discrete element model.

**Figure 2:**
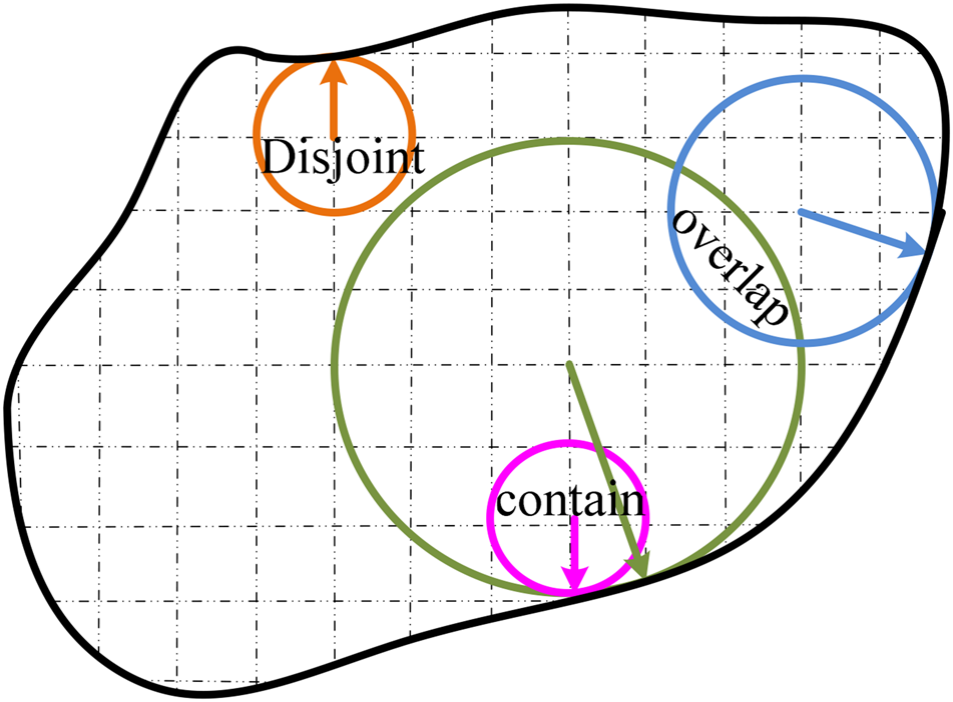
Two-dimensional plane diagram of particle filling relationship.

The shapes of inclusions are similar to ellipsoids, and the surfaces are uneven and irregular. The use of irregular particle modeling technology can better achieve the description of inclusions. Figure 1a shows the inclusion entity, and its 3D solid model is shown in Fig. 1b. The 3D simulation model of the inclusions was meshed, as shown in Fig. 1c, and 77,026 discrete points inside the inclusion particles and 62,068 discrete points outside the surface were obtained with calculation. Taking the inner discrete point as the center and the minimum distance from the inner discrete point to the surface discrete point as the radius, the combination of all spheres generated could describe the shapes of the inclusion particles. The two-dimensional plane diagram is shown in Fig. 2.

It can be seen from Fig. 2 that there had to be a large number of overlaps and containment between circles in the description process of the inclusion shape, which would result in a large number of filled spheres, a large amount of calculation, and a low modeling efficiency. Therefore, a coefficient μ is introduced to remove the overlapped and contained spheres so that the number of spheres could be reduced and the accuracy of the inclusion shape could be ensured. The mechanism can be expressed as follows:

1 $$ d_{nm} + \min \left( {R_{n} ,R_{m} } \right) > \mu \max \left( {R_{n} ,R_{m} } \right) $$

where *d*_*nm*_ is the distance from the center of ball *n* to the center of ball *m*, and min (*R*_*n*_*, R*_*m*_) and max (*R*_*n*_*, R*_*m*_) are the minimum and maximum radii between the *n* and *m* spheres, respectively. When the inequality was established, both the *n* and *m* balls were retained. When the inequality did not hold, the smaller of the *n* and *m* balls was deleted.

Finally, the inclusion discrete element model was formed, as shown in Fig. 1d.

### Filling technology of coal and rock particles simulating multi mineral composition

**Figure 3:**
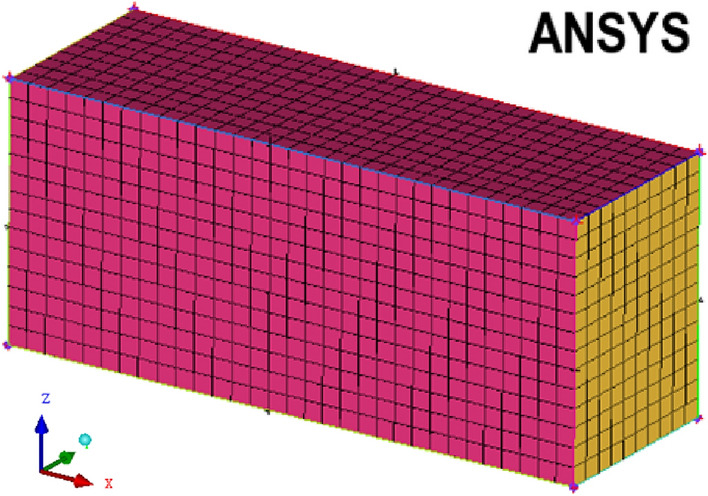
Mesh model.

The internal microstructure of a coal and rock mass is generally composed of a variety of minerals, with complex structures and anisotropy. In order to improve the accuracy of the 3D model of a coal seam, with the help of the secondary development function of the EDEM discrete element, the coal and rock particle filling technology with multi-mineral composition was developed. The 3D modeling software Pro/E was used to establish the spatial model of particle filling. Through the interface between Pro/E and ANSYS, the model was imported into ANSYS for meshing. The mesh model is shown in Fig. 3.

The structure grid shown in Fig. 3 was transformed into an msh file and exported to obtain the X, Y, and Z coordinate position information of particles. Moreover, the properties of the granular materials were defined. The range of the parameter variation was specified. The material parameters obtained with the particles at different positions were different, which broke the fixed value of the coal particle material. With the assumption that the number of particles generated in the space was *n* and the rest time of all particles in the space was *m*, the *i* particles were arranged in chronological order and expressed as follows:

2 $$ P_{it} = \left( {P_{i,1}^{t} ,P_{i,2}^{t} , \cdot \cdot \cdot P_{i,m}^{t} } \right) $$

The material parameters that could represent the properties of the particles were defined as an x series. Then, the particles were arranged in order of position within the range of the stationary time t to construct a matrix (3):

3 $$ PV_{t} \in C^{x \times m} $$

The matrix *PV*_*t*_ was copied *K* times to obtain $PV^{\prime}_{t} \in C^{(x \times K) \times m}$, which was

4 $$ PV_{t}^{^{\prime}} = \left[ {\begin{array}{*{20}c} {PV_{t} } \\ {PV_{t} } \\ \cdot \\ \cdot \\ \cdot \\ {PV_{t} } \\ \end{array} } \right]_{{\left( {x \times K} \right) \times m}} $$

where *K* = *[N/x]* means that by adjusting the size of *K*, the particles would automatically search the position to fill the whole space after randomly matching the material.

**Figure 4:**
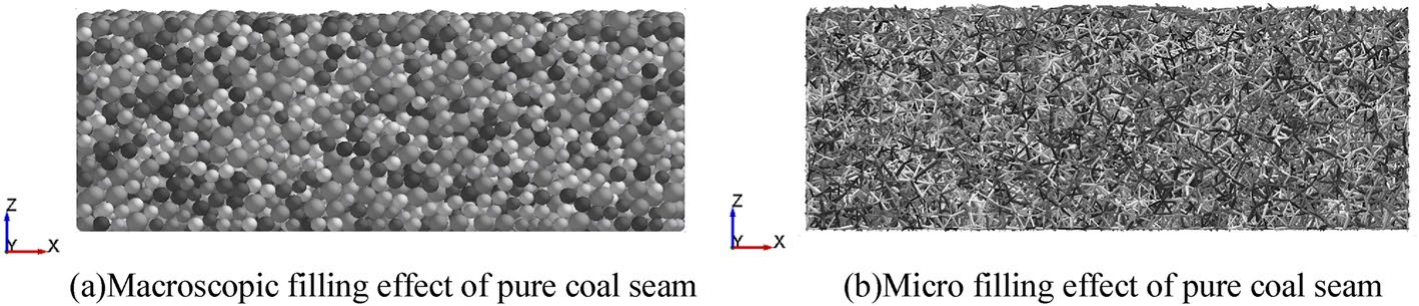
Space filling diagram of pure coal seam.

Taking the pure coal seam as an example, the final coal seam space was as shown in Fig. 4.

### Calculation technology of custom contact model for coal seam working face

#### Custom contact model

**Figure 5:**
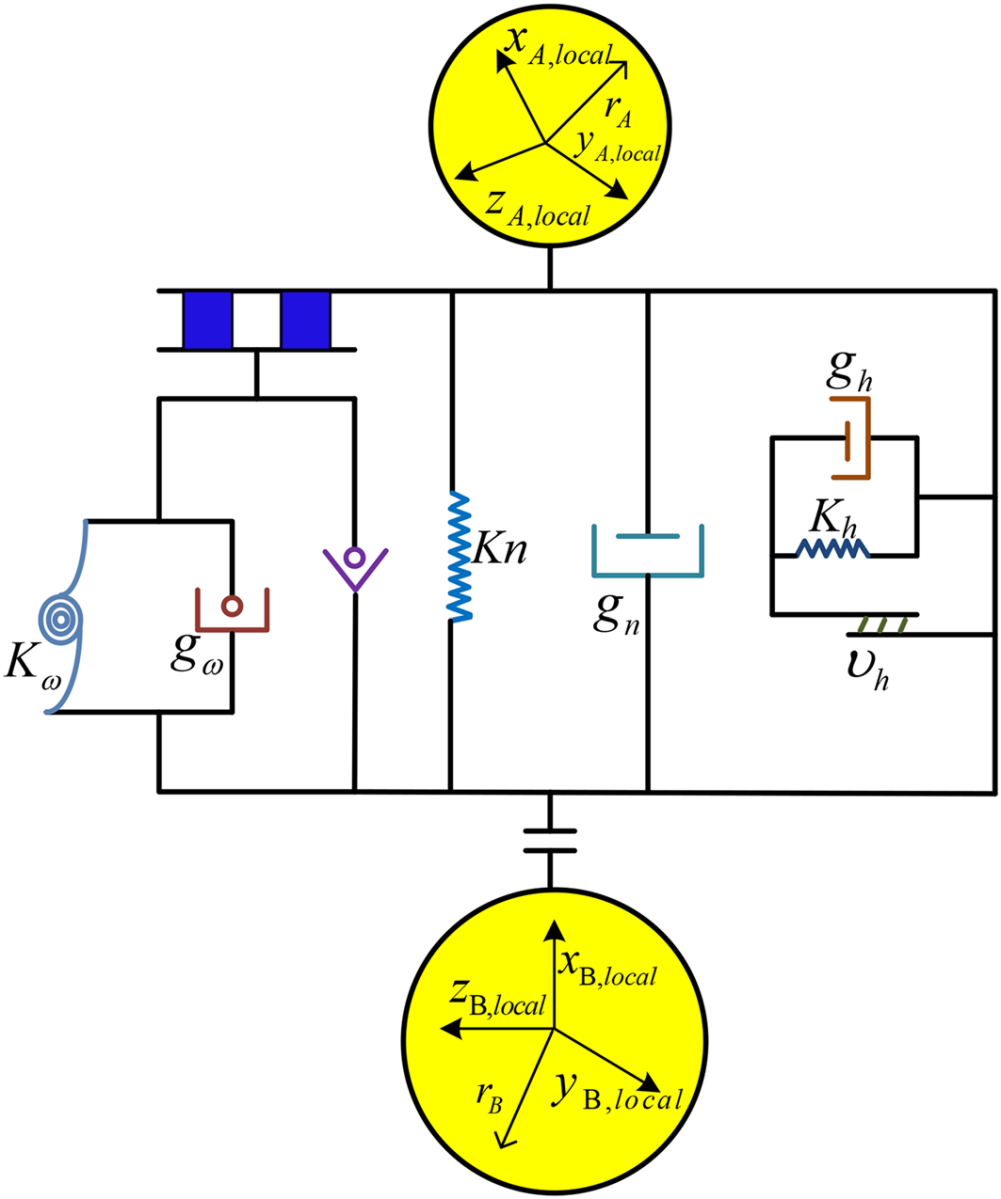
Particles contact model.

The surface of coal and rock is uneven; so, it is difficult to fully reflect the occlusion between particles with only the friction between particles. Therefore, based on the Hertz Mindlin contact model, the torsion force between particles was added, and a custom contact model was established to simulate the surface roughness of coal and rock particles. In the custom contact model, each contact unit was composed of normal, tangential, and rotational contact elements, as shown in Fig. 5.

According to the Hertz theory, the normal contact force in the custom contact model was calculated using the amount of overlap between particles, as follows:

5 $$ F_{n1} = K_{n} S_{n} = \frac{2E}{{3(1 - \mu^{2} )}}(R^{*} )^{\frac{1}{2}} S_{n} $$

6 $$ F_{n2} = - c_{n} \mu $$

7 $$ F_{n} = F_{n1} + F_{n2} $$

where *F*_*n1*_ is the normal elastic contact force on the particles, *F*_*n2*_ is the normal viscous force between particles, *F*_*n*_ is the normal contact force between particles, *K*_*n*_ is the normal stiffness of two particles in contact, *E* is the elastic model of the particles, *μ* is the Poisson's ratio of the particles, R^*^ is the contact radius of particles, *S*_*n*_ is the amount of overlap between particles, and *c*_*n*_ is the viscous damping coefficient.

The tangential contact force between the coal and rock particles was calculated based on the incremental superposition method of the Mindlin theory. Furthermore, tangential sliding friction was introduced in the model. The judgment criterion was based on the Mohr–Coulomb friction law. The mathematical model was expressed as follows:

8 $$ f_{s}^{n + 1} = f_{s}^{n} + K_{s} \mu \Delta t = f_{s}^{n} + \left( {\frac{E}{1 + \mu }} \right)^{\frac{2}{3}} \times \frac{{\left( {12\left( {1 - \mu } \right)R^{*} F_{n} } \right)^{\frac{1}{3}} }}{2 - \mu }\mu \Delta t $$

9 $$ F_{s}^{n + 1} = f_{s}^{n + 1} - c_{s} \mu \begin{array}{*{20}c} {} & {} \\ \end{array} if\left| {m_{r}^{n + 1} } \right| < \mu_{r} R^{*} \left| {F_{n}^{n + 1} } \right| $$

10 $$ F_{s}^{n + 1} = sign\left( {f_{s}^{n + 1} } \right)\mu_{s} \left| {F_{n1}^{n + 1} } \right|\begin{array}{*{20}c} {} & {} \\ \end{array} if\left| {m_{r}^{n + 1} } \right| < \mu_{r} R^{*} \left| {F_{n}^{n + 1} } \right| $$

where *f*_*s*_^*n*+1^ is the predicted value of the tangential force between the particles at time *t*^*n*+*1*^, *f*_*s*_ is the predicted value of the tangential force at time *t*^*n*^, *K*_*s*_ is the tangential stiffness of the two particles in contact, *c*_*s*_ is the tangential viscous damping coefficient of the two particles in contact, *μ*_*s*_ is the tangential static sliding friction coefficient of two particles in contact, and *F*_*s*_^*n*+*1*^ is the contact tangential force between particles at time *t*^*n*+*1*^.

The torsion force between the coal and rock particles was calculated based on the torsion spring rolling mechanics model. Moreover, the selector of Coulomb's law was added to the model to judge whether the contact type between particles is rolling friction. The mathematical model of the anti-rotation bending moment was as follows (11–13):

11 $$ m_{r}^{n + 1} = {\text{m}}_{r}^{n} - K_{r} (\omega_{A} - \omega_{B} )\Delta t $$

12 $$ M_{r}^{n + 1} = m_{r}^{n + 1} - c_{r} (\omega_{A} - \omega_{B} )\begin{array}{*{20}c} {} & {} \\ \end{array} if\left| {m_{r}^{n + 1} } \right| < \mu_{r} R^{*} \left| {F_{n}^{n + 1} } \right| $$

13 $$ M_{r}^{n + 1} = sign(m_{r}^{n + 1} )\mu_{r} R^{*} \left| {F_{n}^{n + 1} } \right|\begin{array}{*{20}c} {} & {} \\ \end{array} if\left| {m_{r}^{n + 1} } \right| < \mu_{r} R^{*} \left| {F_{n}^{n + 1} } \right| $$

where *m*_*r*_^*n*+*1*^ is the estimated value of the anti-rotational bending moment when two coal and rock particles are in contact at time *t*^*n*+*1*^, *m*_*r*_^*n*^ is the estimated value of the anti-rotational bending moment when two coal and rock particles are in contact at time *t*^*n*^, *K*_*r*_ is the rolling torsional stiffness, *ω*_A_ and *ω*_B_ are the rotation speeds of two coal and rock particles in contact with each other, *M*_*r*_^*n*+*1*^ is the anti-rotation bending moment when two coal and rock particles are in contact at time *t*^*n*+*1*^, *c*_*r*_ is the viscous damping coefficient, and *μ*_*r*_ is the static rolling friction coefficient.

The rolling torsional stiffness was determined with the anti-rotation coefficient and the normal contact stiffness. Referring to Jiang's model^20–22^, the calculation process is as follows:

14 $$ K_{r} = \frac{{K_{n} r^{2} \beta^{2} }}{12} $$

where *r* is the average radius of the contact particles, and *β* is the anti-rotation coefficient.

#### Custom energy model

Energy release module was added in the custom contact model through the EDEM/API. In this research, the friction energy and the damping energy released were analyzed after the collision between the coal and rock particles and the shearer drum. The rolling energy consumption release was also analyzed with the rotational contact between particles. This provided judgment data for the intelligent coal and rock identification.

The mathematical model of the energy released by the contact collision is as follows:

15 $$ E_{d}^{l} = \sum\limits_{t = 0}^{t} {\sum\limits_{i = 1}^{N} {\left( {\alpha F_{i} du_{{i_{slip} }} + \alpha M_{i} d\theta_{{i_{slip} }} } \right)} } dt $$

16 $$ E_{d}^{v} = \sum\limits_{t = 0}^{t} {\sum\limits_{i = 1}^{N} {\left( {c_{r} \overline{u}_{i} dx_{{i_{slip} }}^{^{\prime}} + c_{r} \overline{\omega }_{i} d\theta_{{i_{slip} }}^{^{\prime}} } \right)} } dt $$

17 $$ E_{f} = \sum\limits_{t = 0}^{t} {\sum\limits_{i = 1}^{N} {\left( {\overline{F}_{t}^{s} du_{{i_{slip} }}^{s} } \right)} } dt $$

18 $$ E_{r} = \sum\limits_{t = 0}^{t} {\sum\limits_{i = 1}^{N} {\left( {\overline{M}_{i}^{^{\prime}} d\theta_{{i_{slip} }}^{r} } \right)} } dt $$

19 $$ E = E_{d}^{l} + E_{d}^{v} + E_{f} + E_{r} $$

where $E_{d}^{l}$ is the local damping energy, α is the local damping coefficient, $du_{{i_{slip} }}$ is the displacement increment of a single particle, *M*_*i*_ is the unbalanced bending moment of a single particle, $d\theta_{{i_{slip} }}$ is the incremental corner of a single particle, $E_{d}^{v}$ is the viscous damping energy, *c*_*r*_ is the viscous damping coefficient, $\overline{u}_{i}$ is the average velocity of the particles, $dx^{\prime}_{{i_{slip} }}$ is the increment of particle contact displacement, $\overline{\omega }_{i}$ is the average angular velocity of the particles, $d\theta^{\prime}_{{i_{slip} }}$ is the increment of the particle contact corner, *E*_*f*_ is the friction energy, $\overline{F}_{t}^{s}$ is the average tangential force in an incremental time step, $du_{{i_{slip} }}^{s}$ is the increment of plastic sliding displacement, *E*_*r*_ is the rolling energy consumption, $\overline{M}_{i}^{^{\prime}}$ is the average contact bending moment in an incremental time step, $d\theta_{{i_{slip} }}^{r}$ is the plastic rolling displacement increment, and *E* is the total energy released by the contact collision.

#### Model validation

In order to verify the correctness of the custom contact model, four uniform and dense virtual coal specimens were generated with the layered filling method, and the uniaxial compression simulation experiment was carried out. Groups A and B were coal specimens, and groups C and D were rock specimens. Among them, groups B and D were coal and rock specimens with anti-rotation coefficient. The specific parameters are shown in Table 1.

**Table 1:** Parameters of coal and rock samples.

	Density (kg/m^3^)	Elastic modulus (Pa)	Poisson's ratio	Normal contact stiffness (N/m)	Tangential contact stiffness (N/m)	Anti-rotation coefficient
A	1280	2.01 × 10^9^	0.28	1.1098 × 10^8^	8.5104 × 10^7^	0
B	1280	2.01 × 10^9^	0.28	1.1098 × 10^8^	8.5104 × 10^7^	0.7
C	2610	1.83 × 10^10^	0.21	9.7414 × 10^8^	7.4702 × 10^8^	0
D	2610	1.83 × 10^10^	0.21	9.7414 × 10^8^	7.4702 × 10^8^	0.85

	Local damping coefficient	Viscous damping coefficient	Normal viscous damping coefficient	Tangential viscous damping coefficient	Tangential static sliding friction coefficient	Static rolling friction coefficient
A	0.667	0	0.417	0.365	0.672	0.051
B	0.667	0.101	0.417	0.365	0.672	0.051
C	0.346	0	0.803	0.941	0.459	0.124
D	0.346	0.316	0.803	0.941	0.459	0.124

**Figure 6:**
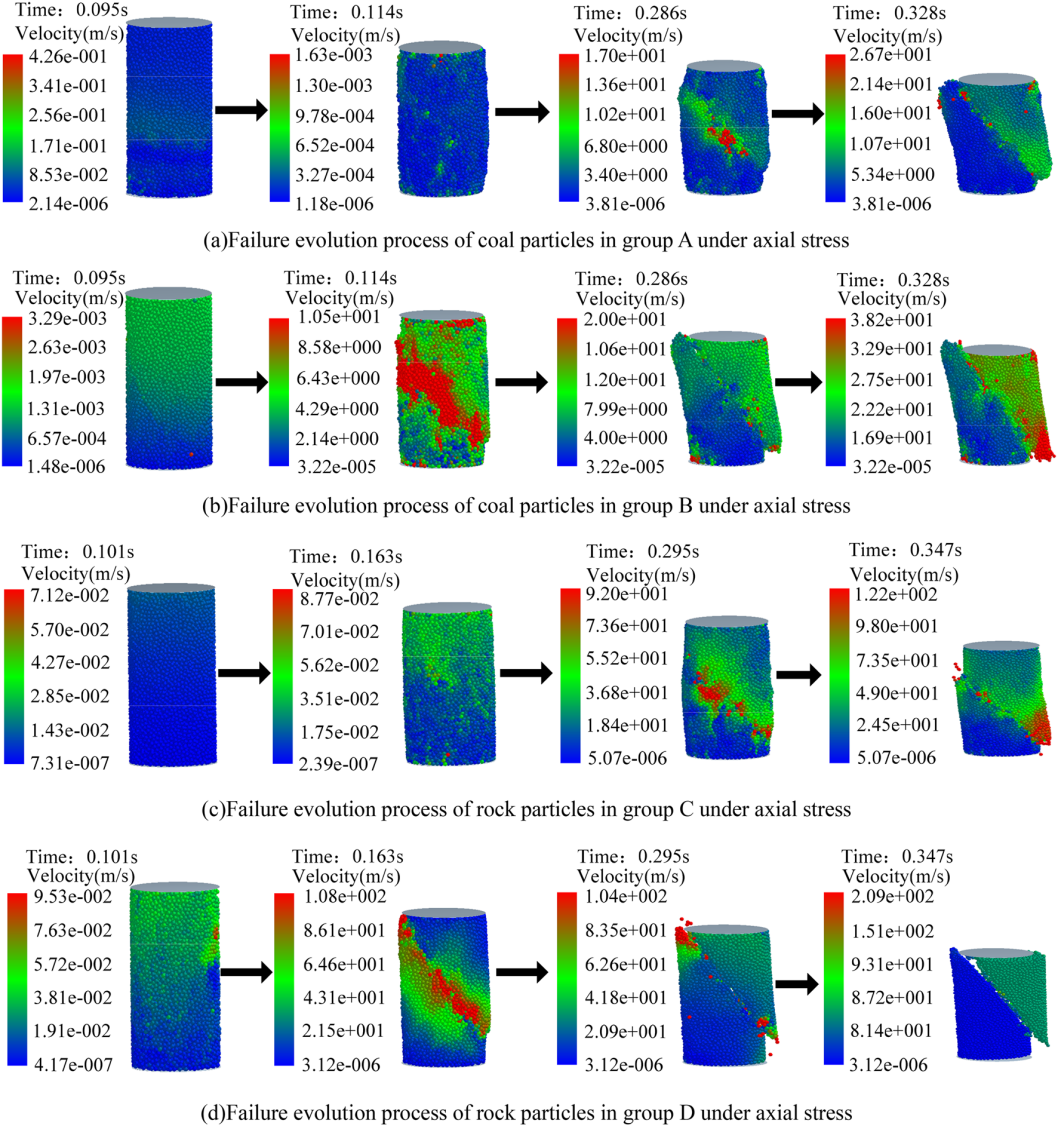
Failure evolution process of coal and rock samples.

The indenter was set to fall at a constant speed of 0.1 m/s. The failure evolution process of the coal and rock specimens was extracted. The coal and rock particles were classified and colored according to the change of their own velocity in the whole process, as shown in Fig. 6.

**Figure 7:**
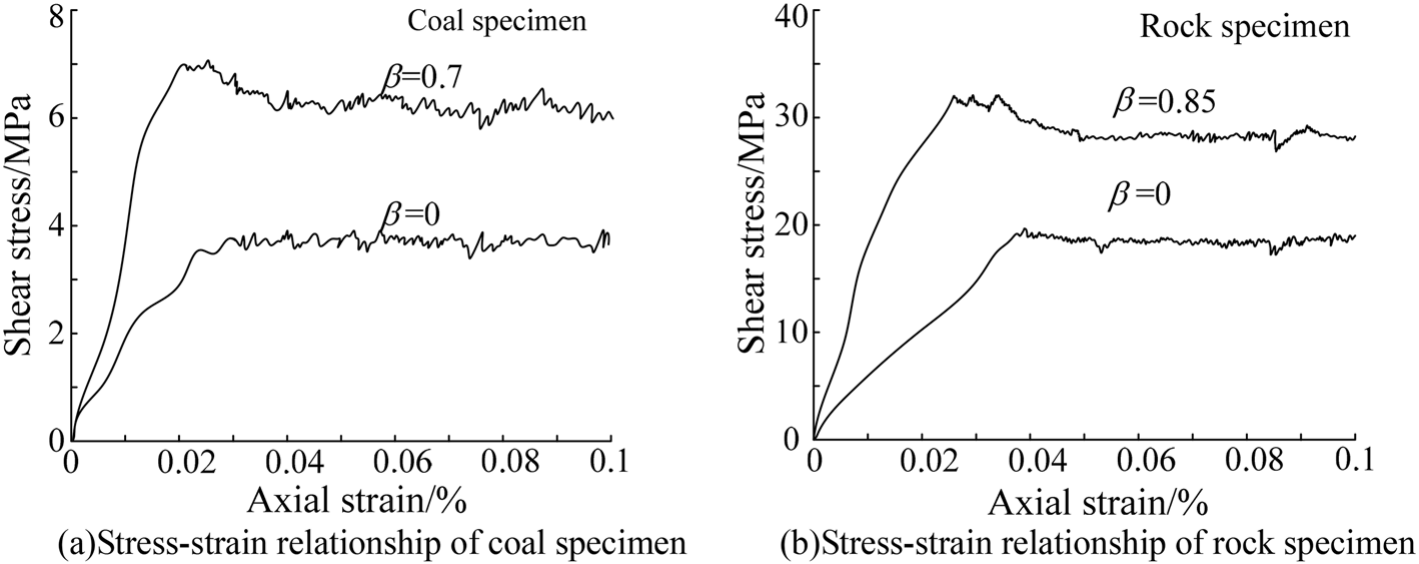
Stress–strain relationship of specimens.

It can be seen from Fig. 6 that the axial deformation of 4 groups of coal and rock specimen was different under the action of indenter. The cracking degree of the coal and rock specimens with the rolling torsion stiffness was greater than that of specimens without rolling torsion stiffness. The stress–strain relationship of the four groups of uniaxial compression specimens during loading was evaluated, and the data were processed based on the MATLAB environment. The stress–strain relationship of the specimens with and without rolling torsion stiffness is shown in Fig. 7.

**Figure 8:**
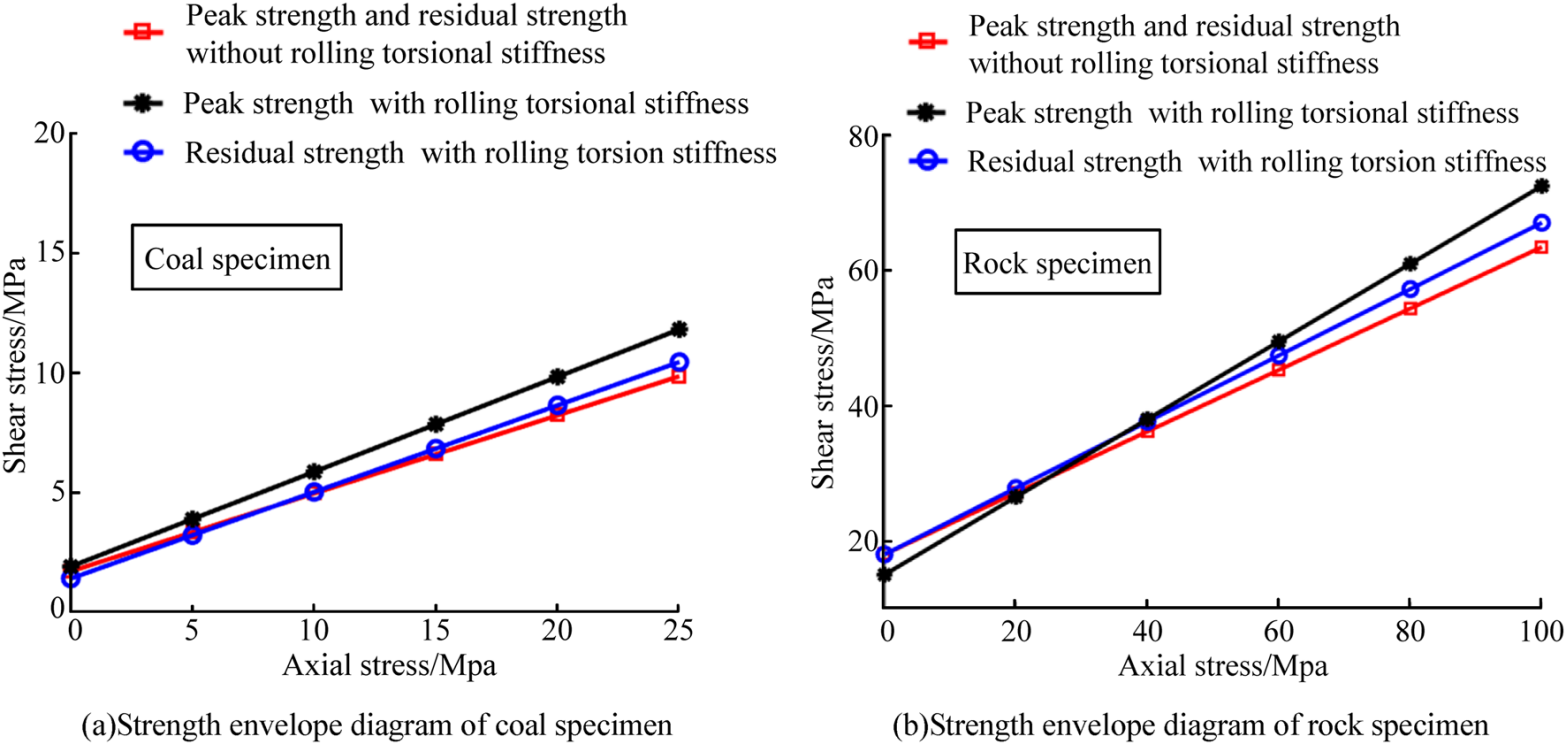
Strength envelope diagram.

It can be seen from Fig. 7 that the peak strength of the coal and rock specimens with rolling torsional stiffness was higher than that of the specimens without rolling torsional stiffness. Moreover, the specimen with rolling torsion stiffness had a stronger elastic deformation ability after the peak value, showing strain softening. The strength envelope diagram of the coal and rock specimens was drawn based on Fig. 7, as shown in Fig. 8.

It can be seen from Fig. 8 that the specimen without rolling torsion stiffness had almost no elastic deformation capacity after reaching the peak strength; so, the peak strength envelope coincided with the residual strength envelope. The line dip angles of the coal and rock specimens without rolling torsion stiffness were 18.08° and 29.47°, respectively. Additionally, according to Moore Coulomb’s law^23^:

20 $$ \sin \varphi = \tan \theta $$

where *θ* is the line dip angle, and *φ* is the internal friction angle. Therefore, the internal friction angles of the coal and rock samples without rolling torsional stiffness could be calculated with formula (20) to be 19.43° and 34.42°, respectively.

Due to strain softening, the peak strength envelope of the specimen with rolling torsional stiffness did not coincide with the residual strength envelope. Therefore, according to Fig. 8, the line dip angles of the residual strength and the peak strength of the coal specimen with rolling torsional stiffness were 19.83° and 21.59°, respectively. Furthermore, according to formula (20), the residual internal friction angle of the coal sample was calculated to be 21.14°. The peak internal friction angle was calculated to be 23.32°. The line dip angles of the residual strength and the peak strength of the rock specimen with rolling torsional stiffness were 30.58° and 32.02°, respectively. In the same way, the residual internal friction angle of the rock specimen was 36.21°, and the peak internal friction angle was 38.72°. The comparison results showed that the internal friction angles of the specimens with rolling torsion stiffness were larger than those of the specimens without rolling torsion stiffness. Therefore, it was proven that the addition of rolling torsion stiffness could improve the ability to simulate the uneven phenomenon for the coal and rock mass, and the feasibility of the custom contact model was verified.

**Figure 9:**
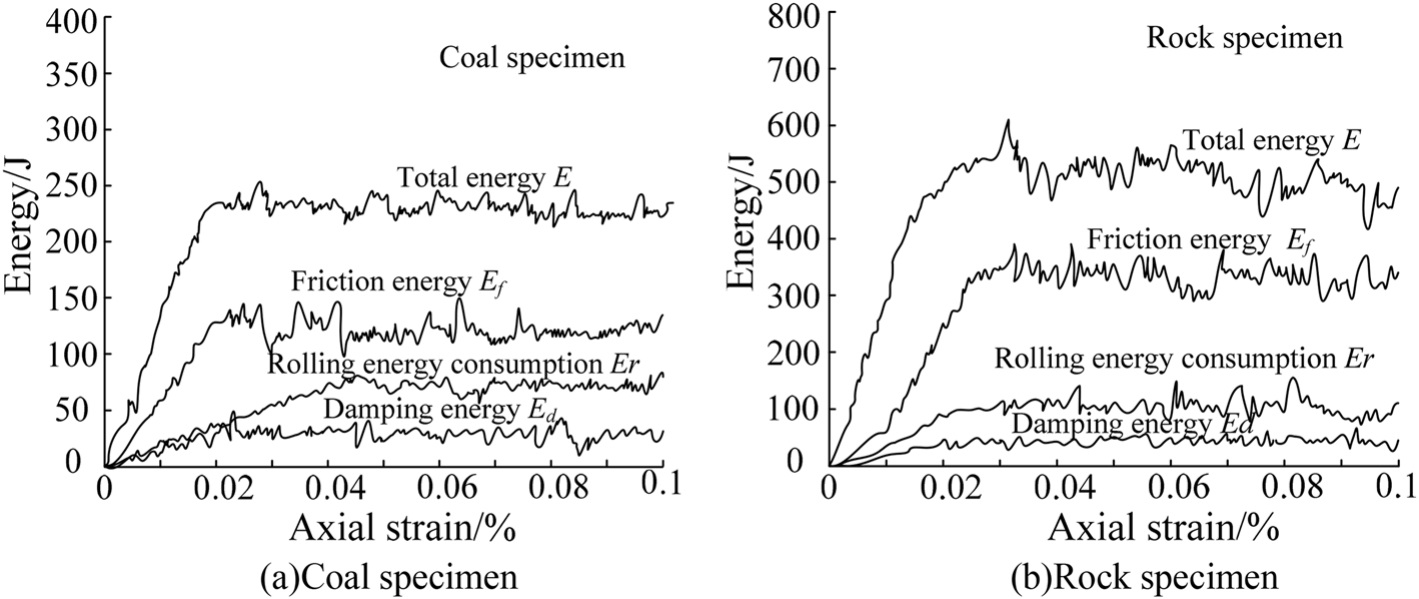
Relationship between dissipated energy and axial strain of specimens.

According to the custom energy module, the data for the energy released during the uniaxial compression test were extracted. Based on MATLAB, the relationship curve between the dissipated energy and the axial strain was obtained, as shown in Fig. 9.

It can be seen from Fig. 9 that the energy released by the specimen with the action of the force reached the peak value with the increase of the axial strain. At this time, the friction energy released by the coal specimen was approximately 1.95 times the rolling energy consumption, and the rolling energy consumption was approximately 2.62 times the damping energy. The friction energy released by the rock specimen was about 3.52 times the rolling energy consumption, and the rolling energy consumption was about 2.13 times the damping energy. After the peak point, the dissipated energy fluctuated around the peak point and tended to be stable. According to the statistics, the average total energy released by the coal specimen was 239 J, and the average total energy released by the rock specimen was 522 J. By comparing the relationship between the dissipated energy of the coal sample and the rock sample, it could be seen that the law of the energy release was closely related to the type of specimen. Therefore, the energy data for the coal and rock material release provided discrimination information for the realization of coal and rock cutting state recognition based on multi-information fusion.

## Test results and simulation analysis

### General situation of geological conditions and distribution of complex coal seams

The average thickness of the 17 layers in the Yanzhou mining area is 1 m, the dip angle of the coal seam is in the range of 5°–13°, the coal seam firmness coefficient is 1.39, the distribution range is wide, and the occurrence is stable. However, the coal seam structure is complex, and it contained iron sulfide nodules. The thickness and the length of the iron sulfide nodules are 100–200 mm and 200–300 mm, respectively. The coal rock firmness coefficient of the nodules is 8.4, and the distribution density is 0.88 a/m^2^^24,25^. The coal seam contained 1–2 layers of gangue, with a thickness of 0.02–0.44 m, and the lithology is carbonaceous sandstone. The roof of the coal seam is limestone with an average thickness of 5.85 m. The floor of the coal seam is aluminous mudstone with an average thickness of 1.17 m.

### Construction of 3D simulation model for geomechanics of complex coal seam

**Figure 10:**
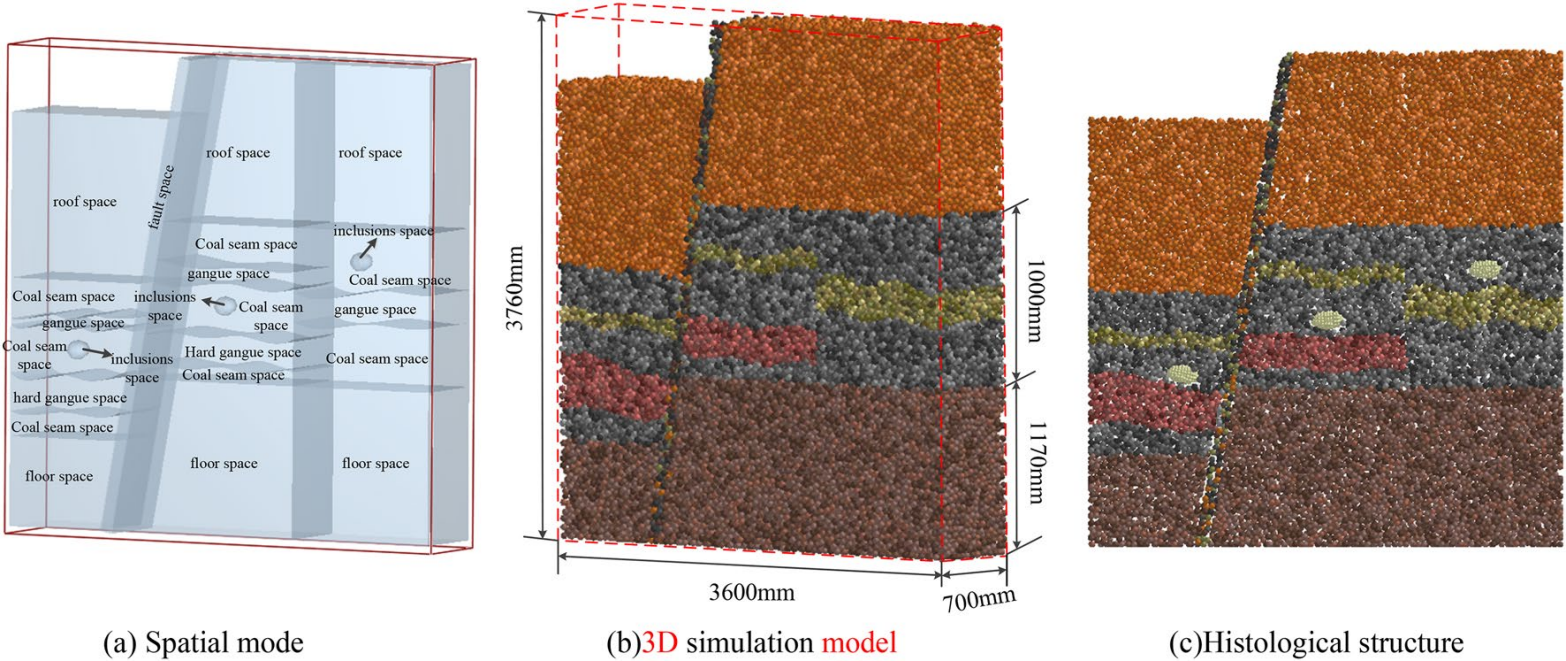
3D simulation model for geomechanics of 17 seam coal layers.

A physical and mechanical properties test and a parameter calibration test were used to obtain the performance parameters of 17 layers in the Yanzhou mining area, and the complex high-precision 3D simulation model for geomechanics of a coal seam was constructed. The solid model was filled in with the simulated fault structure, gangue rock formation, inclusions, roof, and floor according to the occurrence conditions of 17 layers. The final model is shown in Fig. 10. Figure 10a shows the spatial model of the coal seam. According to the spatial model, the specific location of each structure was determined. The 3D simulation model for geomechanics of the coal seam shown in Fig. 10b was formed after particle filling according to the spatial model. Figure 10c shows the internal slice structure of the coal seam. The position and shape of the hard nodule particles in the coal seam could be clearly obtained with the internal slice display of the model.

### Experimental analysis of multi-domain coupling collaborative simulation for coal and rock cutting

**Figure 11:**
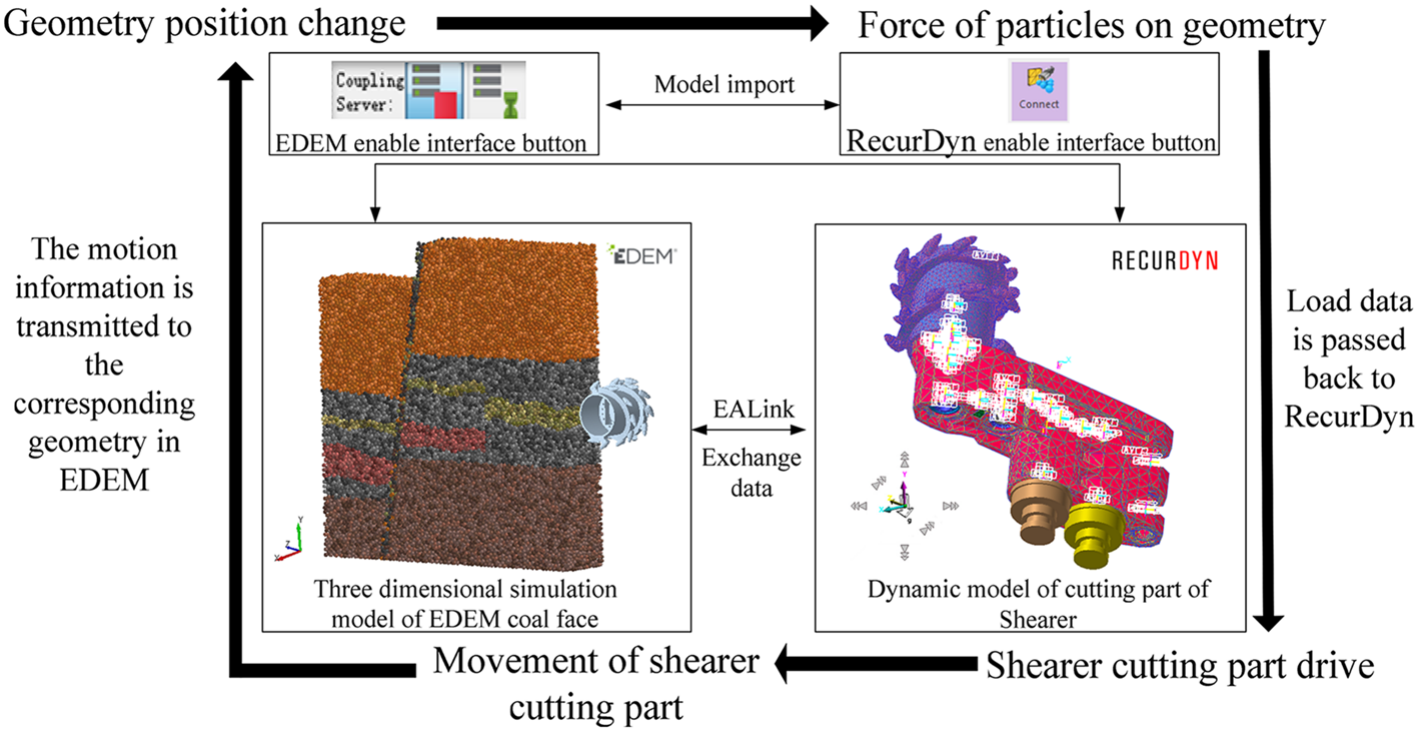
DEM-MFBD interaction process.

According to the test requirements, the dynamic model of the shearer cutting part was constructed, and the 3D solid model of the shearer cutting part was imported into RecurDyn to add the motion pair, contact, and drive. Due to the complex occurrence conditions of the coal seam, in the process of cutting and crushing the coal and rock, the spiral drum was subjected to high impact and nonlinear loads from the coal wall, resulting in a stress concentration and varying degrees of deformation; so, the spiral drum was treated as flexible. Moreover, in order to improve the simulation speed, only the spiral drum of the shearer cutting working mechanism was imported into the EDEM without affecting the experimental results. The 3D simulation model of the DEM-MFBD interaction process is shown in Fig. 11.

**Figure 12:**
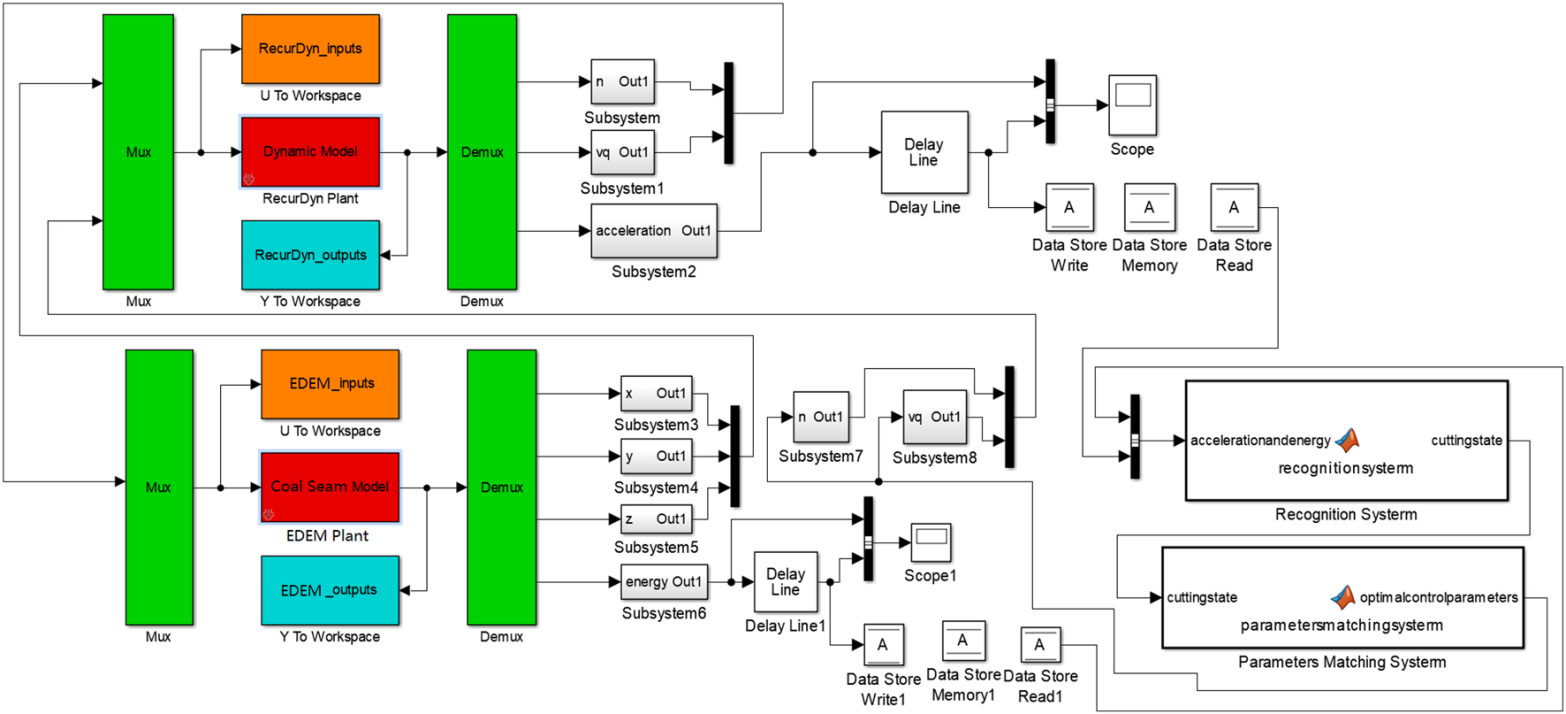
Multi-directional coupling collaborative simulation test platform for coal and rock cutting.

Taking the 3D simulation model for geomechanics of a complex coal seam as the core, combined with the dynamic model of the shearer cutting part, an intelligent multi-directional coupling collaborative real-time simulation platform with multi-source heterogeneous data sharing and mutual feedback was established. In the 3D model of the coal seam, shearer cutting department, intelligent recognition control, and other subsystems, mutual open data sharing was achieved. The real-time interaction process is shown in Fig. 12.

**Figure 13:**
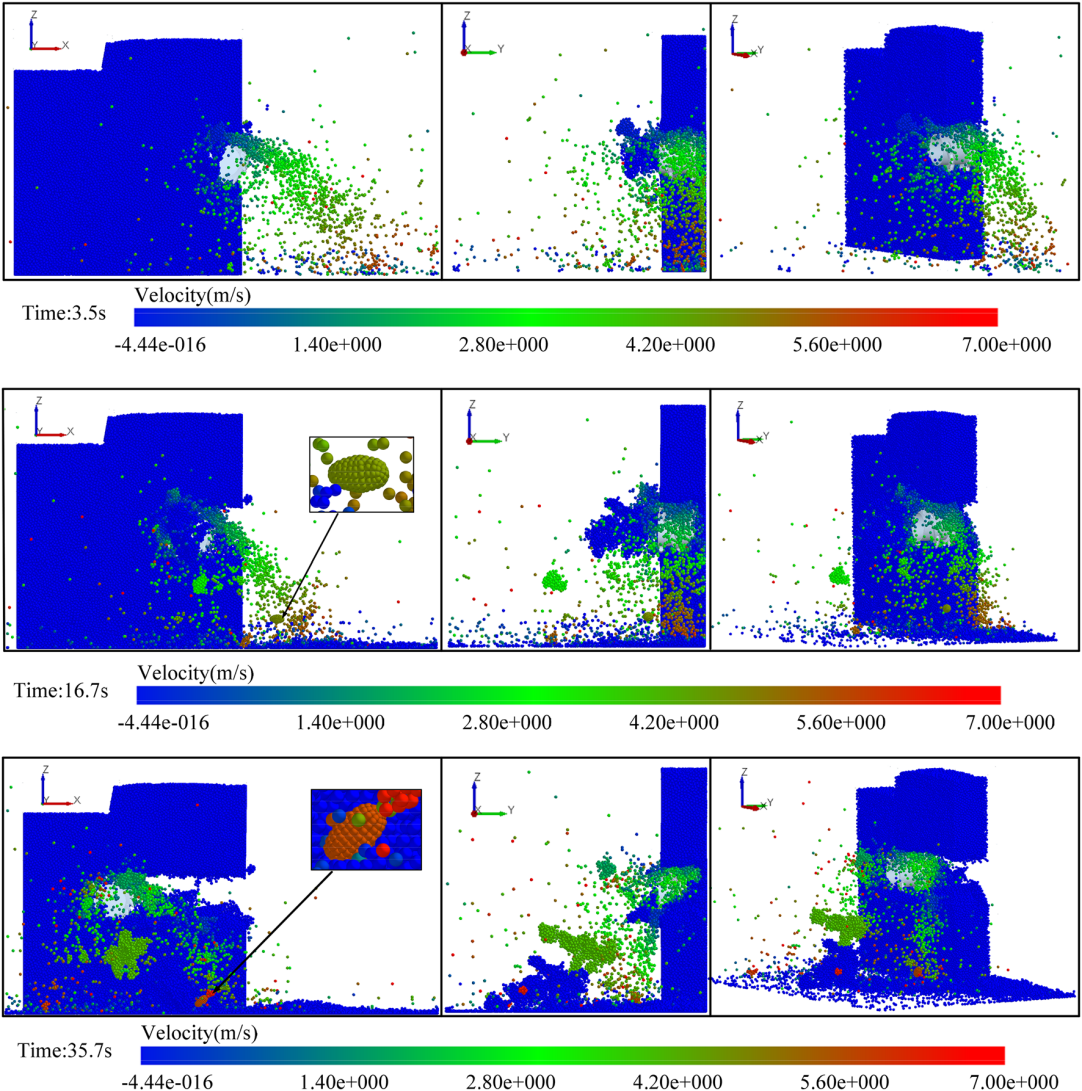
Cutting process of spiral drum.

Using the multi-directional coupling technology of DEM-MFBD-SIMULINK, the translation and rotation motion information of the shearer cutting part dynamic model was transmitted to the corresponding geometry in the cutting complex coal seam model. During the simulation process, the position of the drum cutting complex coal seam was updated in real time, as shown in Fig. 13. With the change of the complex coal seam geological conditions, the change of the drum position affected the position, direction, and size of the force. The force of the coal face on the shearer cutting part was calculated, and the data were sent back to the dynamic model of the shearer cutting part, which achieved the real-time updation of the shearer kinematics and dynamics information.

**Figure 14:**
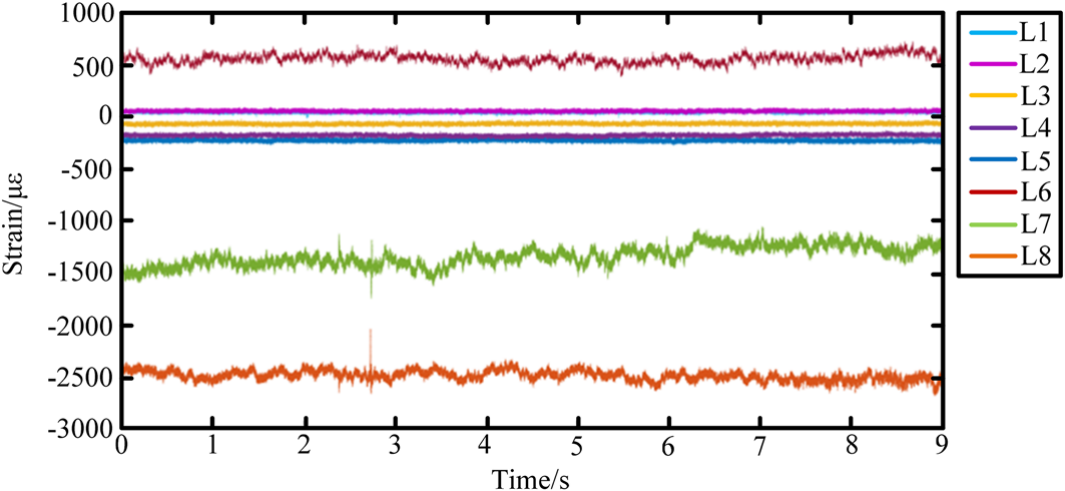
The strain law of the coal and rock at different positions.

During the construction of the high-precision 3D simulation model for geomechanics of the complex coal seam, the bonding between particles caused the force between its structures, which could simulate the stress environment of the real coal and rock mass. Therefore, after the simulation test of the drum cutting of the coal and rock, the strain law of the coal and rock was extracted at different positions in the test process, as shown in Fig. 14. The positions of the coal and rock relative to the drum were denoted as L1, L2, L3, L4, L5, L6, L7, and L8 from far and near. The data results are shown in Table 2. From the average strain value, it could be seen that the farther away from the drum the position was, the smaller the average strain of the coal and rock mass was.

**Table 2:** Statistical results of coal and rock strain data.

Position of coal and rock relative to the drum	Maximum value/$\mu \varepsilon$	Minimum value/$\mu \varepsilon$	Average value/$\mu \varepsilon$	Standard deviation/$\mu \varepsilon$	Root mean square difference/$\mu \varepsilon$	Peak value/$\mu \varepsilon$
L1	86.189	− 13.467	44.607	9.335	45.700	99.656
L2	100.971	15.464	56.086	9.560	56.895	85.507
L3	− 23.461	− 109.874	− 66.489	9.649	67.192	86.458
L4	− 126.962	− 223.090	− 173.534	11.131	173.891	96.128
L5	− 179.057	− 276.643	− 227.581	10.557	227.827	97.586
L6	746.367	384.381	570.233	46.637	572.137	361.984
L7	− 1056.527	− 1747.543	− 1364.901	100.991	1368.632	691.016
L8	− 2048.369	− 3050.494	− 2550.385	78.713	2551.600	1002.125

**Figure 15:**
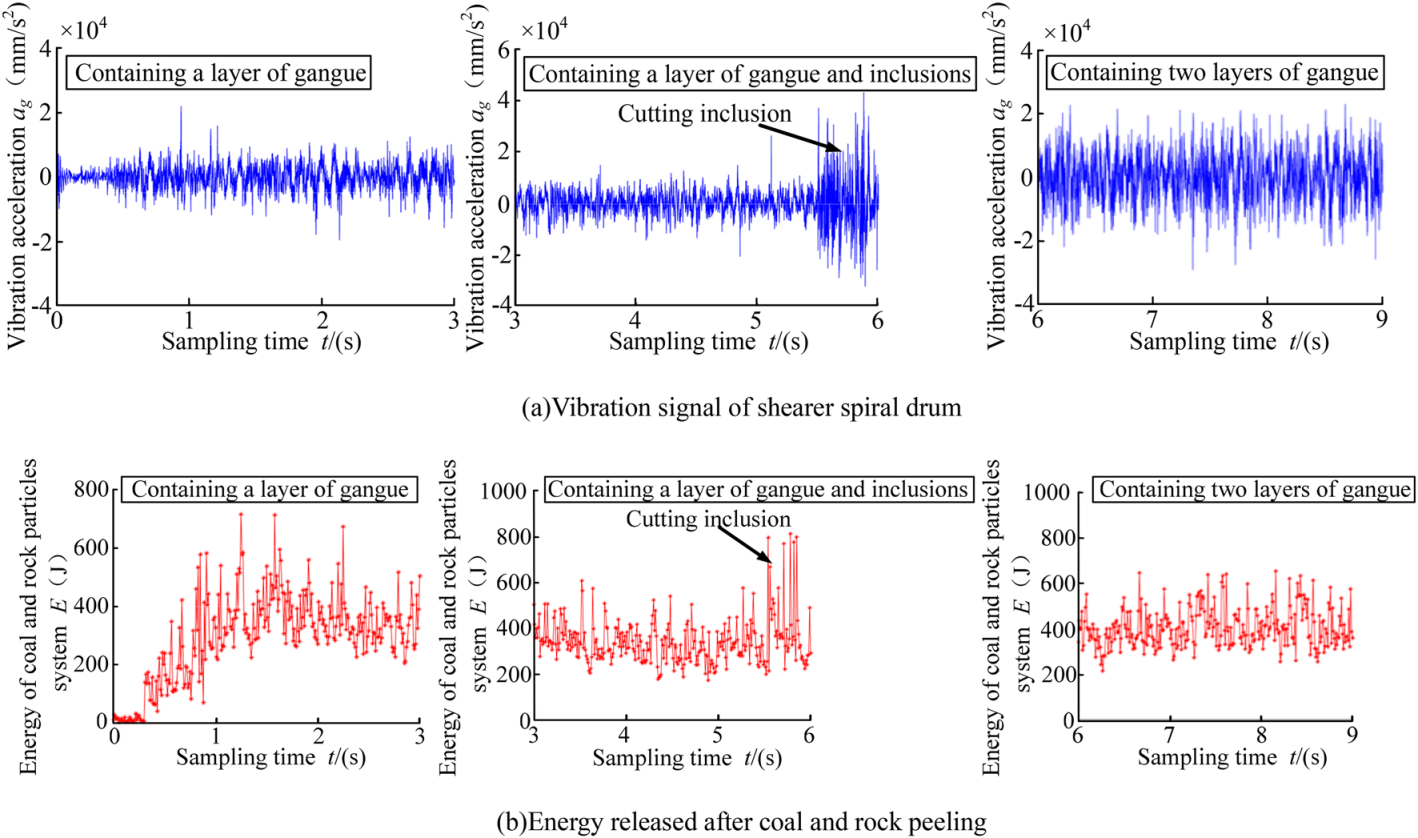
Real-time updated data information.

The constantly updated information data for the multi-directional coupling collaborative real-time simulation platform contained rich geological condition information. Taking the data output interval of 3S as an example, the vibration acceleration of the shearer spiral drum and the energy released after the coal and rock spalling during different time periods were extracted, as shown in Fig. 15.

It can be seen from Fig. 15, with the continuous advance of the shearer cutting part, there were significant differences in the data information during different time periods. The information recognition module could classify these data by processing them in the time domain and the frequency domain. Through the identification and classification of the data, the system could output the types of cutting coal and rock and then output the perceived information to the intelligent control working face to guide the running state of the shearer. The 3D simulation model for geomechanics of the coal seam was applied to the multi-field coupling collaborative real-time simulation experiment of coal and rock cutting, which solved the problem of the real-time transmission of the coal and rock cutting data. In addition, the establishment of the mathematical model for the monitored data was of great significance to optimizing the 3D simulation model for geomechanics of the complex coal seam.

## Experimental verification

**Figure 16:**
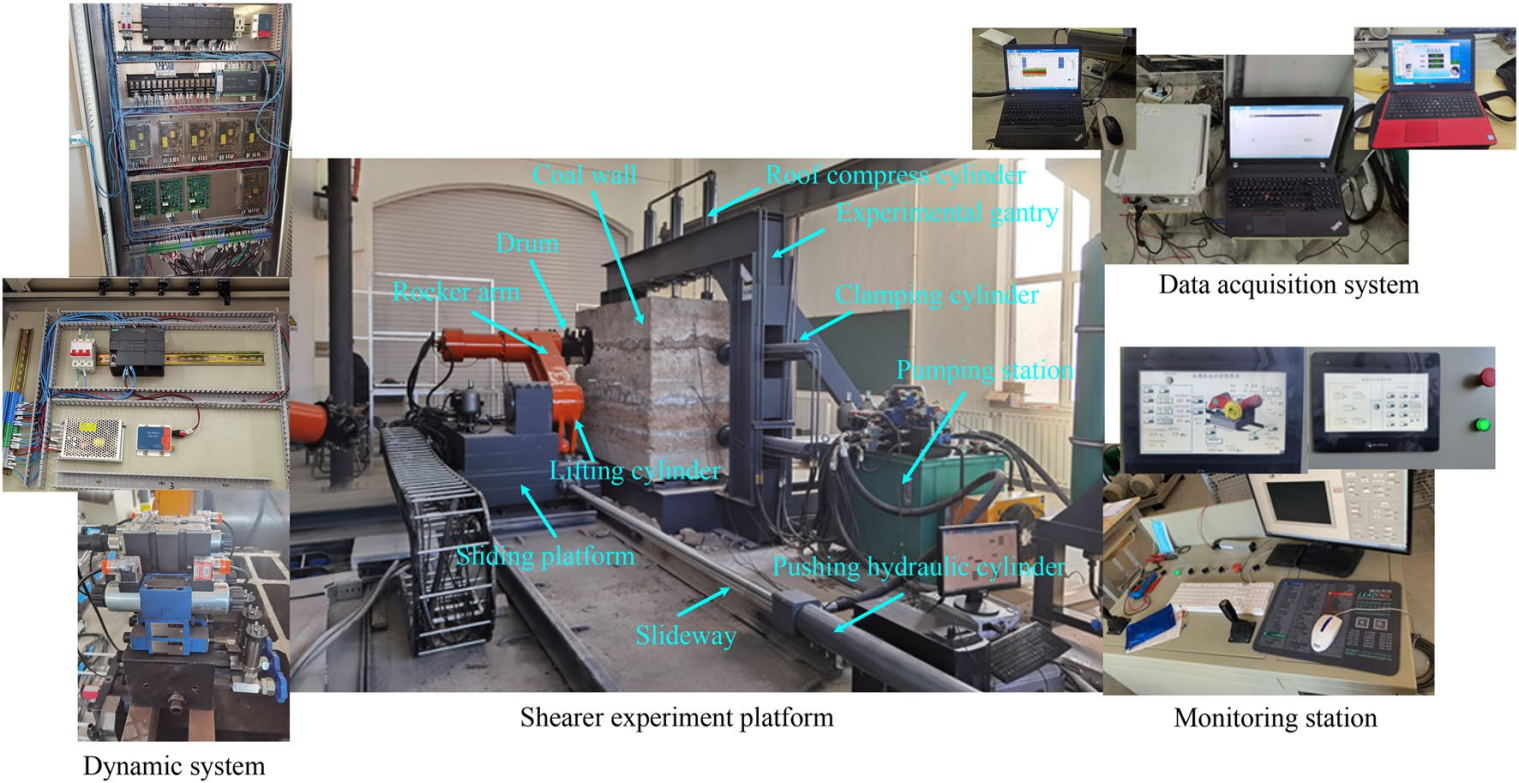
Experimental platform.

In order to verify the correctness of the simulation method, the cutting process of the spiral drum was tested at the experimental center of our group. The construction of the experimental platform was based on the coal breaking theory, the similarity theory, and the similarity coefficient, and the working principle of the shearer was comprehensively considered. The experimental equipment consisted of a coal wall, shearer, power system, and data acquisition system, as shown in Fig. 16.

**Figure 17:**
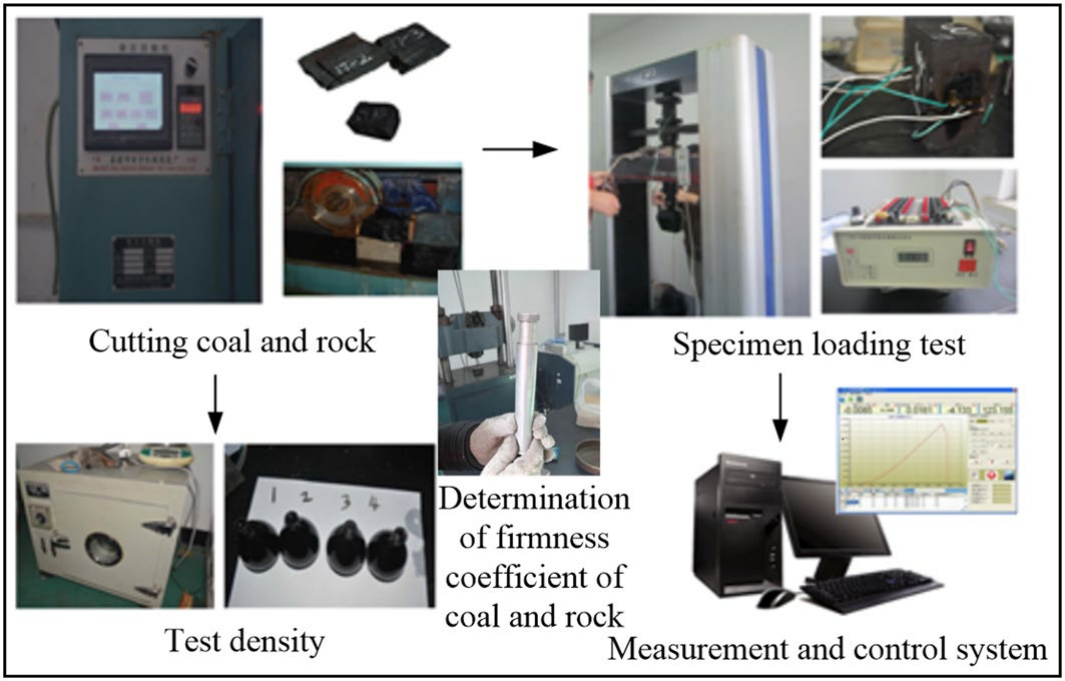
Performance test of coal and rock.

**Figure 18:**
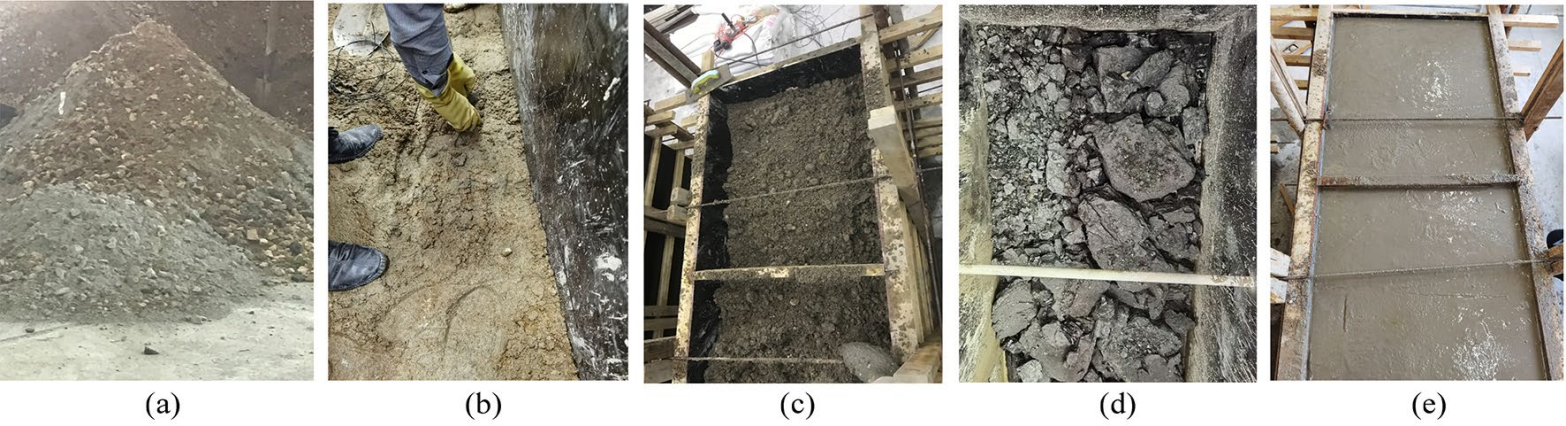
The construction process of the experimental coal wall.

Based on the 17 layers in the Yanzhou mining area, Shandong Province, China, the experimental coal wall was established. Through the cutting coal and rock experiment, specimen loading test, density test experiment and firmness coefficient of coal and rock test experiment shown in Fig. 17, the physical and mechanical parameters of the coal and rock were obtained, and the results are listed in Table 3. The construction process of the experimental coal wall is shown in Fig. 18.

**Table 3:** Coal-rock material parameters.

Material	Density (kg/m^3^)	Elastic modulus (MPa)	Poisson's ratio	Unidirectional strength (MPa)		Internal friction angle(°)	Firmness coefficient
				Compression	Tensile		
Coal	1280	2010	0.28	12	0.3	20.50	1.5
Aluminum mudstone	2460	3620	0.25	34	1.2	33.26	3.5
Limestone	2610	18,300	0.22	52	5.23	38.82	6.8
Hard inclusion	2600	21,500	0.19	64	7.17	42.14	8.4

**Figure 19:**
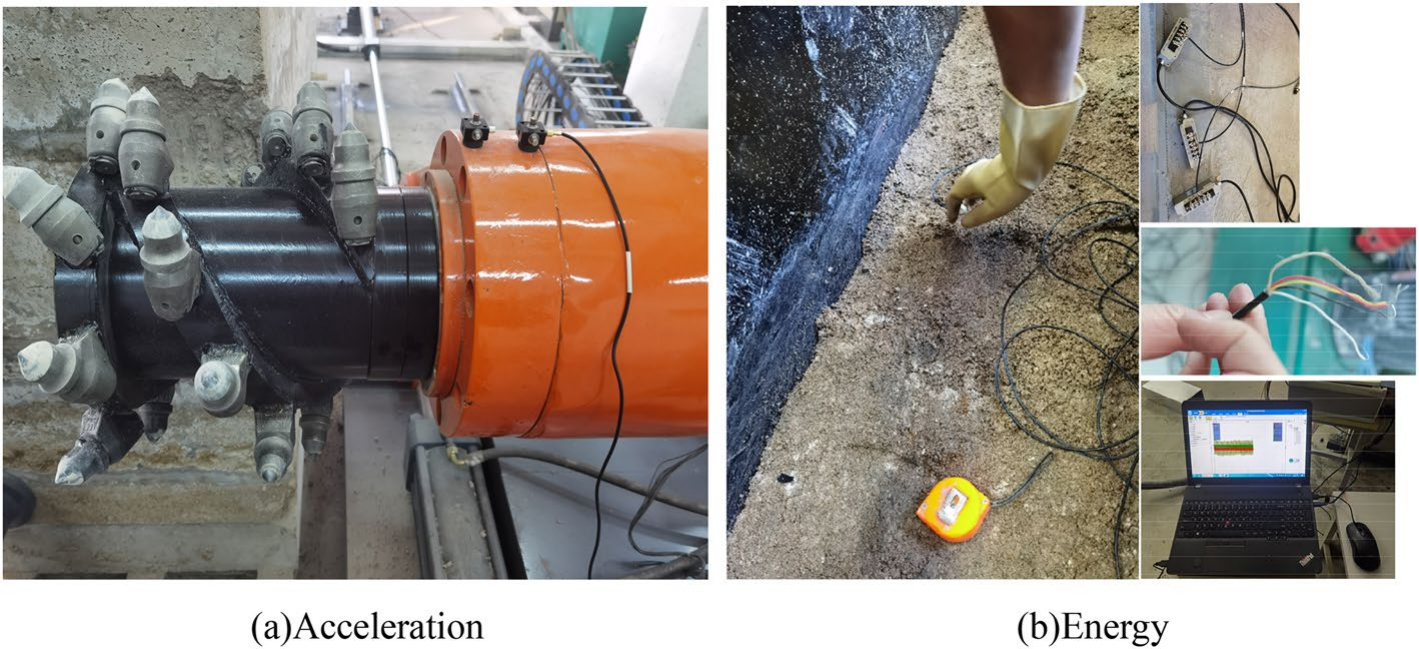
Installation of sensor.

In order to test the vibration signal of the spiral drum, an acceleration sensor was installed at the rear end of the spiral drum, as shown in Fig. 19a. A computer was used for the attitude configuration and the acceleration calibration to make the measurement results more accurate. In order to test the energy released by the coal and rock during cutting, sensors were embedded in the coal wall, as shown in Fig. 19b. The test data collected in the experiment were transmitted to the computer for display, storage, and analysis through the data monitoring equipment.

**Figure 20:**
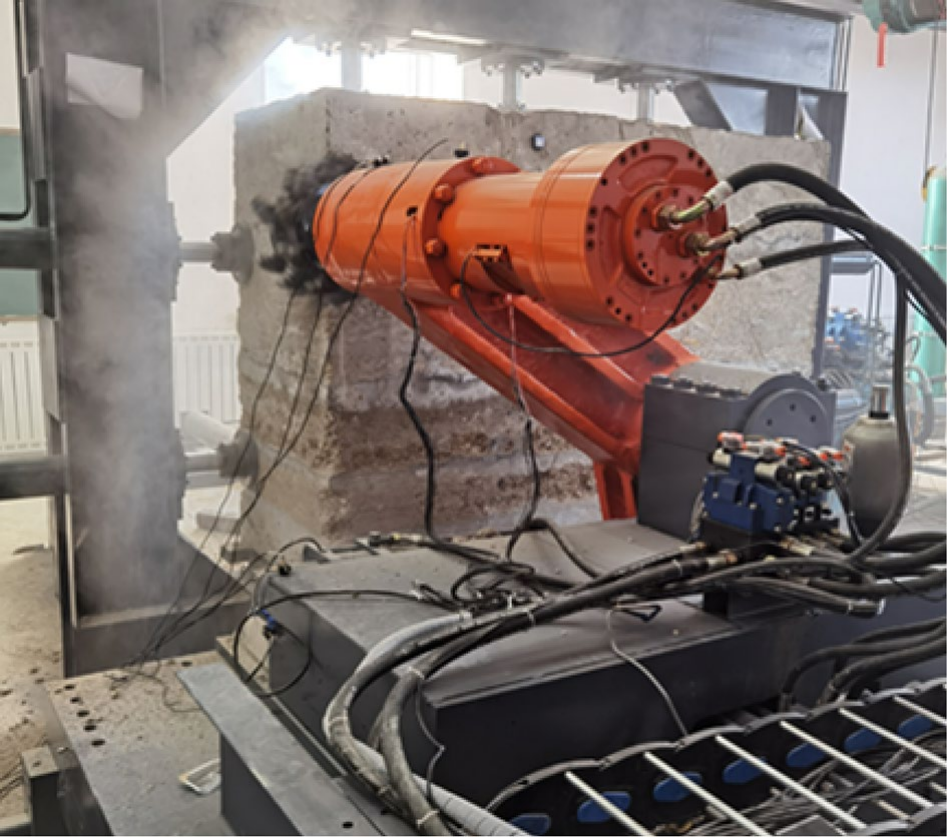
Experimental test process.

Before the test, firstly, the shearer was moved on the guide rail five times to check the running status of the shearer, guide rail, and transfer table. After confirming the normal operation of the system, the pushing cylinder was used to push the shearer to make the pick of the drum contact the coal wall. The cutting depth, traction speed, drum rotation speed, and signal sampling frequency were set to 315 mm, 2.85 mm/s, 141.01 r/min, and 1000 Hz, respectively. The experimental test process is shown in Fig. 20.

**Figure 21:**
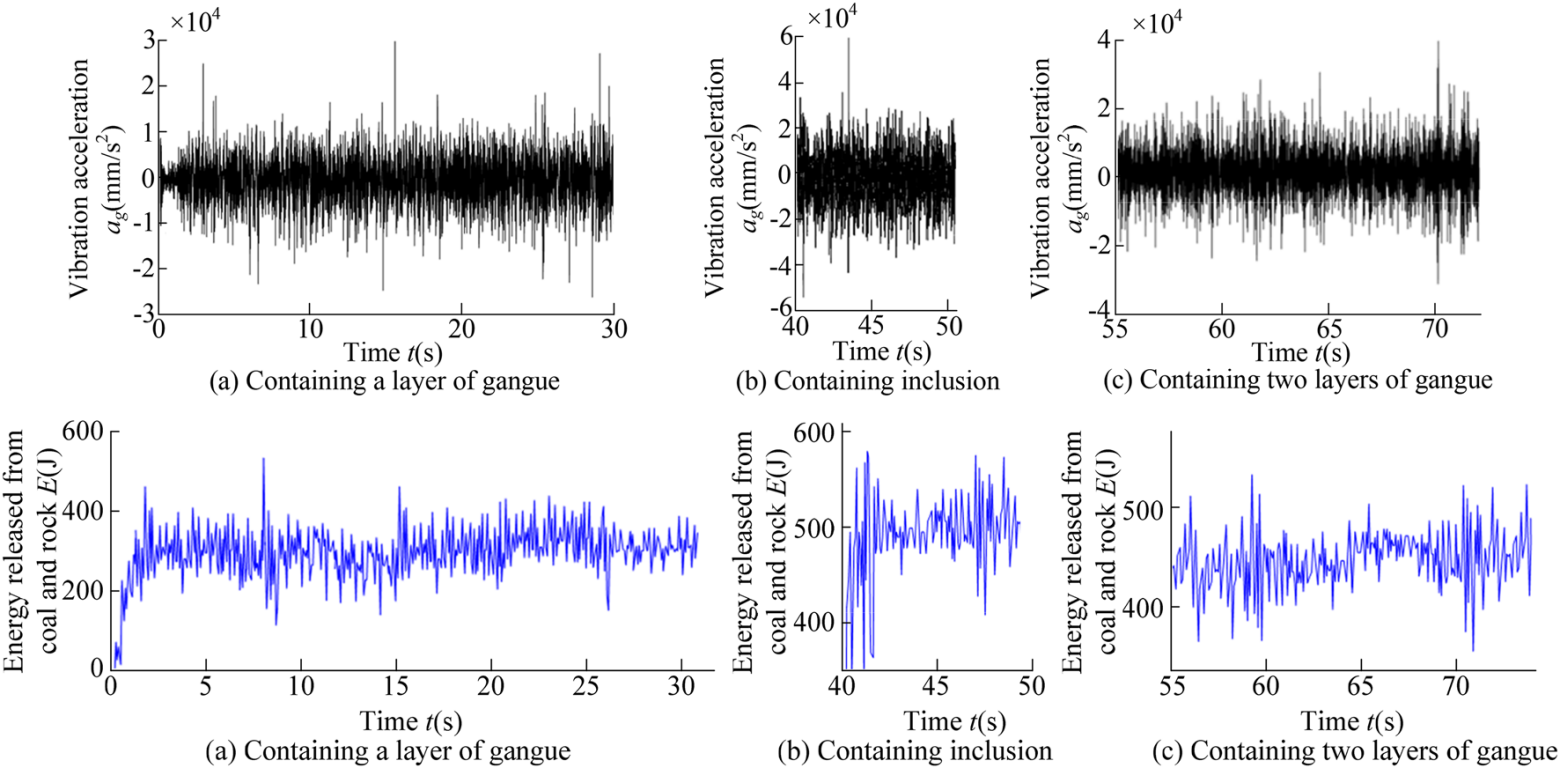
Experimental result.

Through the experimental measurement, the vibration acceleration response signal produced by the drum in the cutting process and the energy released by the cut coal and rock could be effectively obtained. The results are shown in Fig. 21. It can be seen from Fig. 21, when the coal seam cut by the shearer contains a layer of gangue, contains inclusion and contains two layers of gangue, the vibration acceleration of the drum fluctuates in the range of − 20,908.425 to 26,134.872 mm/s^2^, − 53,853.527 to 59,751.179 mm/s^2^, − 33,727.719 to 38,583.154 mm/s^2^ respectively. And the energy released by coal and rock fluctuates in the range of 171.332 to 536.161 J, 357.917 to 589.649 J, 301.075 to 521.473 J respectively. In order to improve the visual contrast of the experimental results, the effective values of the vibration signals of the spiral drum and coal and rock for the released energy with different coal and rock cutting conditions were determined. Moreover, the experimental results were compared with the simulation results to verify the accuracy of the 3D simulation model for geomechanics of the complex coal seam. The comparison between the experimental and simulation results is shown in Table 4.

**Table 4:** Comparison between the experimental and simulation results.

Source	Information	Containing a layer of gangue	Containing two layers of gangue	Containing inclusion
Experimental result	Effective value of vibration acceleration	3947.082 (mm/s^2^)	7527.186 (mm/s^2^)	8136.417 (mm/s^2^)
	Effective value of energy released from coal and rock	321.192 (J)	475.316 (J)	517.221 (J)
The error between the simulation results of the model constructed in this research and the experimental results	Effective value of vibration acceleration	1.10 (%)	1.91 (%)	2.47 (%)
	Effective value of energy released from coal and rock	2.32 (%)	2.18 (%)	3.71 (%)
The error between the simulation results of the traditional model^26^ and the experimental results	Effective value of vibration acceleration	7.06 (%)	10.36 (%)	12.41 (%)
	Effective value of energy released from coal and rock	9.27 (%)	11.57 (%)	12.78 (%)

Since the simulated coal wall was coal and rock with a sticky structure of particles and the experimental coal wall was made by mixing raw materials such as coal, gypsum, and cement, the results had varying degrees of error. In addition, there were many factors in the field test environment, such as the influences of coal dust and water spray dust. This was also the reason for the error between the experimental results and the simulation data. However, it can be seen from Table 4 that for the three working conditions, the error between the numerical value obtained using the coal seam model constructed in this research and the experimental measurement was less than the simulation result for the traditional coal wall model. Therefore, the accuracy of the high-precision 3D simulation model for geomechanics of a complex coal seam was verified. It was proven that the coal seam simulation model constructed in this research was more realistic for obtaining coal and rock information. However, in the actual intelligent cutting process of a shearer, dust and other adverse factors will greatly affect the intelligent mining. Therefore, the dust-removing capacity of a shearer should be improved in an actual mining process, such as using dust-removing water spray and negative pressure dust-removal.

## Conclusion

This paper describes the analysis of the factors that restrict the accuracy of a coal seam 3D simulation model, and the key core technology in the process of modeling is put forward. This technology provides a new idea for optimizing a 3D coal seam model and improves the practicability of a 3D coal seam simulation model.

In the process of building the coal seam 3D simulation model, more information about coal seam geological conditions was integrated, and inclusions, gangue rock formation, fault, roof, and floor structures were added to restore the complexity of the coal seam working face and improve the overall accuracy of the model. The development of the multi-mineral composition of the coal and rock particle filling technology achieved the random diversification of the coal seam particle materials. Considering the uneven surface of the coal and rock particles, an anti-rotation factor was added between the particles. The feasibility of the custom contact model was verified with experiments.

Through the multi-directional coupling collaborative real-time simulation experiment of coal and rock cutting, it was proved that the 3D simulation model for geomechanics of the coal seam could be applied to the modules of the coal seam spatial analysis and coal rock cutting state recognition. The problem of insufficient data integration was solved, and the real-time transmission of the coal and rock cutting data was realized. Furthermore, this provided an effective guarantee for research on the high-efficiency cutting of an intelligent shearer.
